# Cross-Reactive Neuraminidase Antibodies Afford Partial Protection against H5N1 in Mice and Are Present in Unexposed Humans

**DOI:** 10.1371/journal.pmed.0040059

**Published:** 2007-02-13

**Authors:** Matthew R Sandbulte, Gretchen S Jimenez, Adrianus C. M Boon, Larry R Smith, John J Treanor, Richard J Webby

**Affiliations:** 1 Department of Infectious Diseases, St. Jude Children's Research Hospital, Memphis, Tennessee, United States of America; 2 Vical, San Diego, California, United States of America; 3 Infectious Diseases Unit, University of Rochester, Rochester, New York, United States of America; National Institutes of Health, United States of America

## Abstract

**Background:**

A pandemic H5N1 influenza outbreak would be facilitated by an absence of immunity to the avian-derived virus in the human population. Although this condition is likely in regard to hemagglutinin-mediated immunity, the neuraminidase (NA) of H5N1 viruses (avN1) and of endemic human H1N1 viruses (huN1) are classified in the same serotype. We hypothesized that an immune response to huN1 could mediate cross-protection against H5N1 influenza virus infection.

**Methods and Findings:**

Mice were immunized against the NA of a contemporary human H1N1 strain by DNA vaccination. They were challenged with recombinant A/Puerto Rico/8/34 (PR8) viruses bearing huN1 (PR8-huN1) or avN1 (PR8-avN1) or with H5N1 virus A/Vietnam/1203/04. Additional naïve mice were injected with sera from vaccinated mice prior to H5N1 challenge. Also, serum specimens from humans were analyzed for reactivity with avN1. Immunization elicited a serum IgG response to huN1 and robust protection against the homologous challenge virus. Immunized mice were partially protected from lethal challenge with H5N1 virus or recombinant PR8-avN1. Sera transferred from immunized mice to naïve animals conferred similar protection against H5N1 mortality. Analysis of human sera showed that antibodies able to inhibit the sialidase activity of avN1 exist in some individuals.

**Conclusions:**

These data reveal that humoral immunity elicited by huN1 can partially protect against H5N1 infection in a mammalian host. Our results suggest that a portion of the human population could have some degree of resistance to H5N1 influenza, with the possibility that this could be induced or enhanced through immunization with seasonal influenza vaccines.

## Abstract 2

Humoral immunity against endemic human H1N1 influenza viruses can partially protect mice against H5N1 challenge, raising the possibility that a portion of the human population could have some degree of resistance against avian flu.

## Editors' Summary

**Background.:**

Every winter, millions of people catch influenza—a viral infection of the airways. Most recover quickly but influenza can kill infants, elderly people, and chronically ill individuals. To minimize these deaths, the World Health Organization recommends that vulnerable people be vaccinated against influenza every autumn. Annual vaccination is necessary because flu viruses continually make small changes to the viral proteins (antigens) that the immune system recognizes. Each year's vaccine contains disabled versions of the circulating strains of influenza A type H1N1 and H3N2 viruses, and of influenza B virus. The H and N refer to the major influenza A antigens (hemagglutinin and neuraminidase), and the numbers refer to the type of each antigen; different H1N1 and H3N2 virus strains contain small variations in their respective hemagglutinin and neuraminidase type. Vaccines provide protection against seasonal influenza outbreaks, but sometimes flu viruses emerge that contain major antigenic changes, such as a different hemagglutinin type. These viruses can start pandemics (global outbreaks) because populations have little immunity to them. Many scientists believe that avian (bird) H5N1 influenza virus (which has caused about 250 confirmed cases of human flu and 150 deaths) could trigger the next human pandemic.

**Why Was This Study Done?:**

Avian influenza H5N1 virus has not started a human pandemic yet because it cannot move easily between people. If it acquires this property, it could kill millions before an effective vaccine could be developed, so researchers are looking for other ways to provide protection against avian H5N1. One possibility is that an immune response to the human type 1 neuraminidase (huN1) in circulating H1N1 influenza virus strains and vaccines could provide some protection against avian H5N1 influenza virus, which contains the closely related avian type 1 neuraminidase (avN1). In this study, the researchers have investigated this possibility in mice and in a small human study.

**What Did the Researchers Do and Find?:**

The researchers immunized mice with DNA encoding the huN1 present in a circulating H1N1 virus. They then examined the immune response of the mice to this huN1 and to avN1 from an avian H5N1 virus isolated from a human patient (A/Vietnam/1203/04). Most of the mice made antibodies (proteins that recognize antigens) against huN1; a few also made detectable levels of antibodies against avN1. All the vaccinated mice survived infection with a man-made flu virus containing huN1, and half also survived infection with low doses of a man-made virus containing avN1 or A/Vietnam/1203/04. To test whether the antibodies made by the vaccinated mice were responsible for this partial protection, the researchers collected serum (the liquid part of blood that contains the antibodies) from them and injected it into unvaccinated mice. Again, about half of the mice survived infection with the H5N1 virus, which indicates that the huN1-induced immunity against H5N1 is largely mediated by antibodies. Finally, the researchers tested serum samples from 38 human volunteers for their ability to inhibit neuraminidase from an H1N1 virus and two H5N1 viruses (antibodies to neuraminidase reduce viral replication and disease severity by inhibiting neuraminidase activity). Most of the sera inhibited the enzyme from the H1N1 virus; and seven also inhibited the enzyme from both H5N1 viruses.

**What Do These Findings Mean?:**

These findings indicate that a vaccine containing huN1 induces the production of antibodies in mice that partly protect them against H5N1 infection. In addition, the human study suggests that some people may have some degree of resistance to H5N1 influenza because of exposure to H1N1 viruses or routine influenza vaccination. These results, while intriguing, don't show that there is actual protection, but it seems well worth doing additional work to address this question. The researchers also suggest that many more people might have been infected already with H5N1 but their strong H1N1 immunity meant they had only mild symptoms, and this hypothesis also deserves further investigation. Overall, these findings raise the possibility that seasonal influenza vaccination may provide some protection against pandemic H5N1. It is worth discussing whether, even while further studies are underway, seasonal vaccination should be increased, especially in areas where H5N1 is present in birds.

**Additional Information.:**

Please access these Web sites via the online version of this summary at http://dx.doi.org/10.1371/journal.pmed.0040059.

- A related *PLoS Medicine* Perspective article by Laura Gillim-Ross and Kanta Subbarao is available
- US Centers for Disease Control and Prevention provides information about influenza for patients and professionals, including key facts about avian influenza and vaccination
- US National Institute of Allergy and Infectious Disease has a feature on seasonal, avian and pandemic flu
- World Health Organization has fact sheets on influenza and influenza vaccines, and information on avian influenza
- UK Health Protection Agency provides information on seasonal, avian, and pandemic influenza

## Introduction

The ongoing spread of highly pathogenic avian H5N1 influenza virus across Eurasia and Africa has led to over 200 confirmed infections in humans, more than half of whom have died [1]. In the event that an H5N1 strain gains the ability to spread efficiently from human to human, the lack of subtype-specific immunity would make the human population highly vulnerable. Indeed, the occurrence of pandemic influenza relies on a lack of immunity to the virus in a large proportion of the human population.

Currently licensed seasonal influenza vaccines are designed to protect humans from the prevailing strains of human influenza A lineages H1N1 and H3N2 and of influenza B virus. These vaccines' principal target is the most abundant viral surface antigen, hemagglutinin (HA). Immunization against this sialic acid receptor–binding glycoprotein typically elicits neutralizing antibodies, which may act by blocking the attachment of the virus to host-cell receptors or by interfering with HA-mediated viral fusion [2,3]. The annually determined trivalent influenza vaccines are standardized on the basis of the content of their HA components. Neuraminidase (NA), the other major surface protein and determinant of serotype, is not standardized in current vaccines, meaning that the amount is likely to vary from batch to batch. Antibodies against NA do not block infection, but they can inhibit the enzymatic activity of NA [4,5]. Therefore, immunization against NA can decrease viral replication in the lungs and reduce disease severity upon subsequent challenge [4–7]. Although HA and NA are both highly immunogenic, intact influenza virions reportedly induce a humoral response skewed toward HA because of antigenic competition [8]. However, immunization with a commercial trivalent subvirion vaccine in which the HA and NA components are dissociated increases NA-specific antibody titers in humans [9].

Although the NA of avian H5N1 influenza virus strains (avN1) and of human H1N1 strains (huN1) are classified in the same serotype, phylogenetic differences between the two lineages are considerably greater than those within each lineage. Despite clear public health implications, it remains unknown whether antibodies raised against huN1 can inhibit replication of H5N1 influenza virus or mediate any degree of protection from H5N1 infection. Standard trivalent human influenza vaccines regularly require substitution of the H1N1 component to optimize protection against new prevailing strains. This fact suggests that cross-lineage protective immunity is improbable, particularly in the case of H1N1 versus H5N1, of which HA genes have low homology. Still, even limited cross-reactivity between H5N1 virus and pre-existing huN1-specific antibodies or T cells might reduce viral replication and disease, with significant implications for H5N1 infection in the human population.

## Methods

### Viruses and Vaccine

A/Hong Kong/213/03 (H5N1) and A/Vietnam/1203/04 (H5N1) influenza viruses were obtained from the World Health Organization collaborating laboratories. A/New Caledonia/20/99 (H1N1) influenza virus was obtained from the virus repository of Dr. Robert Webster (St. Jude Children's Research Hospital, Memphis, Tennessee, United States). Gene segments of A/Vietnam/1203/04, A/New Caledonia/20/99, and A/Puerto Rico/8/34 (PR8) (H1N1) influenza viruses were cloned into plasmids for virus rescue and gene reassortment by the 8-plasmid reverse genetics method, as described previously [10,11]. Viruses so derived were propagated in the allantoic cavities of 10-d-old embryonated chickens' eggs. These reassortant viruses include “PR8-huN1,” which bears the NA gene segment of human-lineage A/New Caledonia/20/99 and seven complementary gene segments of PR8, and “PR8-avN1,” which bears the NA gene segment of avian-lineage A/Vietnam/1203/04 and seven complementary gene segments of PR8. A DNA vaccine was constructed by cloning the nucleotide sequence of the open reading frame of the NA gene segment of A/New Caledonia/20/99 (huN1) into the plasmid backbone VR10551. Expression of this sequence was under the control of a human cytomegalovirus promoter [12].

### Animals and Experimental Design

BALB/cJ mice were purchased from The Jackson Laboratory (http://www.jax.org) and housed in the Animal Resources Center at St. Jude Children's Research Hospital. Mice (aged 7 wk) received intramuscular injections of huN1 DNA vaccine (100 μg), control diluent, or plasmid lacking a gene insert (100 μg) at weeks 0 and 3. Serum was collected from orbital bleeds taken before secondary vaccination and before viral challenge. At week 6, under Animal Biosafety Level 3 enhanced conditions, mice were anesthetized with avertin and inoculated intranasally with challenge viruses. PR8-huN1, PR8-avN1, and A/Vietnam/1203/04 were administered at doses of 10 and 100 50% MLD_50_ (mouse lethal doses). Body weights were monitored regularly and deaths noted daily. Individual animals showing obvious hind limb paralysis were euthanized humanely. To achieve passive immunization, 11-wk-old BALB/cJ mice were intraperitoneally injected with 350 μl of serum collected from above-mentioned mice as follows. HuN1-immune serum was pooled from huN1 DNA-vaccinated mice 17–20 d after administration of dose 2. Positive control serum was pooled from mice that survived challenge with A/Vietnam/1203/04 (H5N1) virus, and negative control serum was pooled from saline-injected mice. Recipient mice were challenged with 10 MLD_50_ of A/Vietnam/1203/04 18 h after passive immunization.

### Serology

Sera from huN1-immunized mice were treated with receptor-destroying enzyme and analyzed by ELISA for specificity to PR8-huN1 and PR8-avN1 viral antigens. These viral antigens were propagated in 10-d-old embryonated chickens' eggs, concentrated, purified over sucrose gradients, and pelleted by ultracentrifugation. Microtiter plates were coated with PR8-huN1or PR8-avN1 (6 μg/ml in suspension) by overnight incubation at 4 °C. After plates were washed six times with PBS containing 0.05% Tween 20 (PBS-Tween) and nonspecific antibody binding was blocked by incubation for 2 h with PBS-Tween with 10% fetal bovine serum; sera were added in a series of doubling dilutions. Overnight incubation at 4 °C was followed by washing with PBS-Tween, incubation with alkaline phosphatase-conjugated goat anti-mouse IgG (Southern Biotech [http://www.southernbiotech.com]) for 2 h at room temperature, further washing with PBS-Tween, and addition of the substrate para-nitrophenylphosphate. Absorbance at 405 nm was measured with an ELISA plate reader, and endpoint titers were defined as the serum dilution at which the signal strength was twice that of strain-matched control serum. The lower limit of IgG detection was an endpoint titer of 20. Serum samples were collected from 38 human volunteers. These samples were assayed for NA-specific antibodies by a standard NA inhibition method [13]. In brief, the neuraminidase activity of each virus was standardized according to colorimetric analysis of sialic acid release from fetuin substrate. Inhibition titers were determined by the reduction of this activity after virus incubation with serially diluted serum, when compared with control reactions lacking serum. NA inhibition titers were measured against A/New Caledonia/20/99, and reassortant PR8 viruses containing the HA and NA of A/Hong Kong/213/03, or A/Vietnam/1203/04. The HA of the latter two viruses was manipulated to remove the polybasic amino acids associated with high virulence. This allowed the handling of these viruses at Biosafety Level 2 conditions.

### Statistical Analysis

Statistical analysis of differences in mortality between experimental and control treatment groups was performed using a one-sided Fisher's exact test. Statistical significance was designated for differences with *p*-values less than or equal to 0.05. IgG titers measured by ELISA were log_2_ transformed, and mean titers from the two treatment groups were compared by Student's t test. Samples lacking detectable IgG (detection limit 20) were assigned a titer of 10 for statistical calculations.

## Results

**Figure 1:**
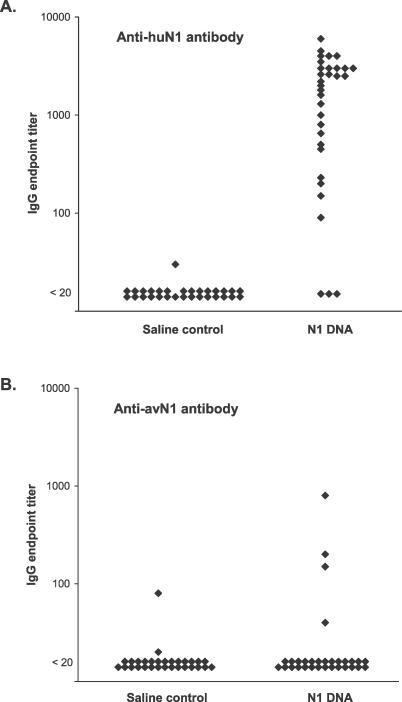
NA-Specific Antibody Responses to Immunization Serum was collected from BALB/cJ mice after two injections with plasmid encoding the NA of human H1N1 influenza strain A/New Caledonia/20/99 (huN1) or saline only. Antibody reactivities with huN1 and the NA of H5N1 influenza strain A/Vietnam/1203/04 (avN1) were determined by ELISA. (A) Between the huN1 DNA and saline-only treatment groups there was a statistically significant difference in mean IgG titers against huN1 (*p* < 0.001). (B) IgG titers detected against avN1 were not significantly different between the treatment groups (*p* = 0.092). The antibody titer against each target was defined as the reciprocal of the highest serum dilution that produced an ELISA signal twice as intense as the signal from equivalently diluted naïve serum. The lower limit of detection was a titer of 20.

The aim of the present study was to determine whether immunity to huN1 provides sufficient cross-reactive immunity to avN1 to confer resistance to H5N1 influenza virus. To this end, we vaccinated female BALB/cJ mice twice with a plasmid encoding the NA of A/New Caledonia/20/99 (H1N1) to induce NA-specific immunity. A/New Caledonia/20/99 is the source of H1N1 antigen for currently available trivalent influenza vaccines. Readily detectable antibody responses to the homologous huN1 antigen were induced in nearly all mice, as shown by ELISA (Figure 1A). In equivalent sera from control mice, titers of antibodies to huN1 were essentially undetectable, with a mean significantly different from huN1-immunized mice (*p* < 0.001). Using an equivalent ELISA, we also tested samples for antibody reactivity with NA of A/Vietnam/1203/04 (H5N1), which shares 80% amino acid identity with NA of A/New Caledonia/20/99. Reactivity with the heterologous avN1 was detected only in a small proportion of serum samples of the huN1 DNA vaccinated group (4/32) or the control mice (2/30) (Figure 1B), and the difference in mean titers was statistically insignificant (*p* = 0.092). Sera from three mice in the huN1 DNA vaccinated group had avN1-specific titers greater than 100, whereas no sample from the control group possessed this level of reactivity.

**Figure 2:**
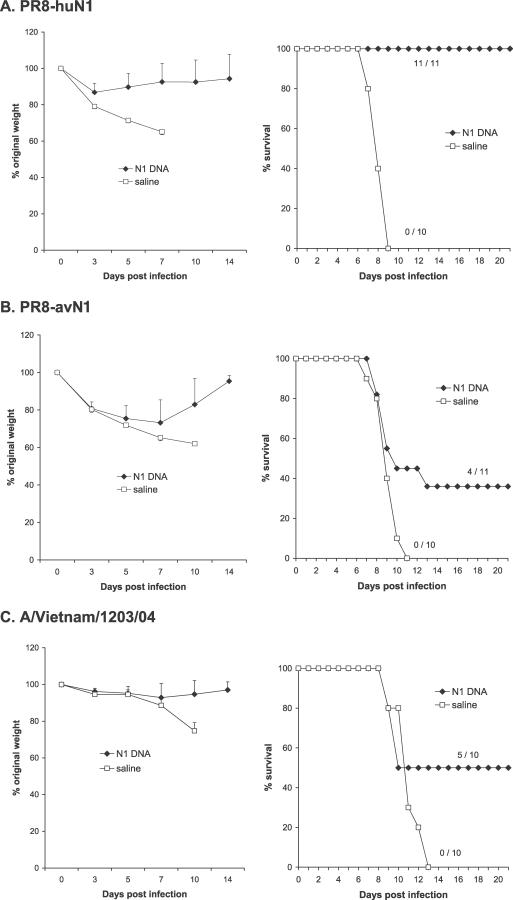
Challenge of huN1-Immunized Mice with Influenza Viruses Possessing Homologous or Heterologous NA Genes Mice immunized twice with huN1 DNA or saline alone (control) were inoculated intranasally with PR8-huN1 (A), PR8-avN1 (B), or A/Vietnam/1203/04 (C). Mean weight change and survival data are shown. Differences in survival between huN1 DNA-vaccinated and saline control groups were statistically significant for PR8-huN1 challenge (*p* < 0.001) and A/Vietnam/1203/04 challenge (*p* = 0.016), and approached but did not reach significance for PR8-avN1 challenge (*p* = 0.055). Error bars represent standard deviation values. Statistical comparisons are by Fisher's exact test.

Mice vaccinated with huN1 DNA demonstrated robust resistance to challenge with 10 MLD_50_ of PR8-huN1 (Figure 2A). Although all control mice lost a substantial amount of weight and died from this challenge infection, all of the vaccinated animals survived and largely recovered the weight they lost early in the infection. Protection of vaccinated mice against mortality from the homologous virus was highly significant (*p* < 0.001), which demonstrates the vaccine's potency. Challenge with 10 MLD_50_ of PR8-avN1 was also 100% lethal to control mice, whose disease course was similar to that in mice infected with PR8-huN1 (Figure 2B). In addition, challenge with PR8-avN1 at this dose caused greater than 20% mean weight loss by day 7 in the mice vaccinated with huN1 DNA. However, a subset of vaccinated mice (4/11) regained weight and survived, evidence that partial cross-protection resulted from the immune response to huN1. The difference in mortality between immunized and control groups upon heterologous PR8-avN1 challenge approached but did not reach statistical significance (*p* = 0.055). All mice vaccinated with huN1 DNA were protected from mortality upon a 100 MLD_50_ challenge dose of PR8-huN1 (*p* < 0.0001), but cross-protection against heterologous PR8-avN1 at this 10-fold higher dose was weak (2/10 survival, *p* = 0.26, unpublished data).

The third viral challenge with H5N1 strain A/Vietnam/1203/04 was highly lethal, as previously reported [11,14,15]. Infection with 10 MLD_50_ of A/Vietnam/1203/04 killed all control mice (Figure 2C). Disease resulting from infection with this H5N1 virus was more prolonged than that caused by the PR8-based challenge viruses, and the infected naïve mice showed a somewhat biphasic pattern of weight loss often accompanied or followed by hind leg paralysis. In contrast, mice vaccinated against huN1 typically had more moderate weight loss and fewer of them showed neurological signs. Half (5/10) of the vaccinated mice recovered from challenge with the H5N1 influenza virus, which demonstrates statistically significant protection from mortality (*p* = 0.016). Against a 10-fold higher challenge dose of H5N1 virus (100 MLD_50_) vaccination with huN1 DNA failed to protect against mortality (unpublished data).

**Figure 3:**
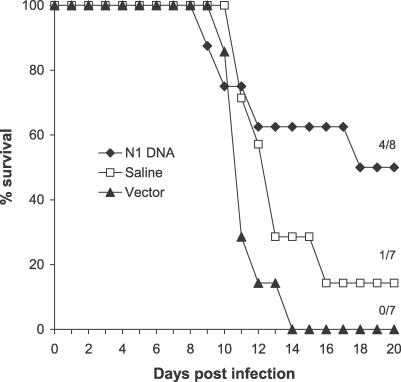
Sequence Dependence of huN1 DNA Vaccine Induced Protection against Lethal H5N1 Infection Mice vaccinated at week 0 and week 3 with huN1 DNA (*n* = 8), saline (*n* = 7), or empty plasmid vector (*n* = 7) were challenged with 10 MLD_50_ of A/Vietnam/1203/04 (H5N1) at week 6. Survival was monitored daily. Although the difference in mortality between saline-injected and huN1 DNA–vaccinated groups did not reach statistical significance (*p* = 0.18), the difference in mortality between huN1 DNA vaccinated mice and those given empty vector DNA approached significance (*p* = 0.051). Statistical comparison is by Fisher's exact test.

In an additional experiment we addressed the possibility that the protection conferred by huN1 DNA against heterologous challenge was a result of nonspecific stimulation of the innate immune system, such as by CpG DNA motifs. Along with mice receiving huN1 DNA recombinant plasmid, a group of control mice received only the backbone plasmid without a gene insert. Vaccination with the empty vector failed to protect any animals against the lethal effects of A/Vietnam/1203/04 (Figure 3). Owing to small treatment groups in this experiment, the difference in mortality between mice receiving huN1 DNA (4/8) versus saline (1/7) was not statistically significant (*p* = 0.18), but the difference between huN1 DNA and empty vector treatment (0/7) approached significance (*p* = 0.051). Thus, we exclude nonspecific immune stimulation by plasmid DNA as the mechanism for cross-subtype protection.

**Figure 4:**
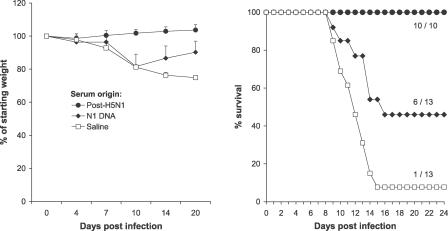
Cross-Protective Effects of huN1-Immune Serum against H5N1 Influenza Mice were passively immunized with serum from mice that had recovered from H5N1 influenza virus infection (post-H5N1), were vaccinated twice with huN1 DNA (N1 DNA), or were vaccinated twice with saline alone. Eighteen hours after passive immunization, recipient mice were challenged with 10 MLD_50_ of A/Vietnam/1203/04. Changes in mean weight (left graph) and survival (right graph) were monitored over 24 d. The difference in survival between recipients of serum from huN1 DNA-vaccinated and saline control mice was statistically significant (*p* = 0.037). Error bars represent standard deviation values. Statistical comparison is by Fisher's exact test.

We hypothesized that protection from H5N1 influenza by huN1 immunization is mediated by the humoral immune response. To test this hypothesis, we pooled sera from mice injected twice with huN1 DNA (prechallenge), from mice injected with saline only, or from mice that had recovered from infection with H5N1 influenza virus, and we transferred the sera intraperitoneally to naïve mice before challenging them with 10 MLD_50_ of A/Vietnam/1203/04. Serum from survivors of infection protected all recipient mice from severe disease and death upon homologous challenge, whereas mice that received serum from saline-injected animals were highly susceptible to this challenge (1/13 survival) (Figure 4). In comparison, serum from huN1 DNA–vaccinated mice was partially protective: 46% (6/13) of recipient mice survived challenge with A/Vietnam/1203/04, which represents a statistically significant difference from naïve serum (*p* = 0.037). This outcome closely mirrors that of the vaccinated animals themselves. This set of results demonstrates that huN1-induced immunity against H5N1 virus is mediated, at least in large part, by a humoral response. The considerable weight loss of the passively immunized mice that survived challenge in this experiment might reflect a contribution by cell-mediated immunity to cross-lineage protection. Alternatively, the discrepancy in recovery may be attributable to the dilution of immune serum in passively immunized mice, the lack of memory B cells in these mice, or stress resulting from the serum transfer procedure.

**Figure 5:**
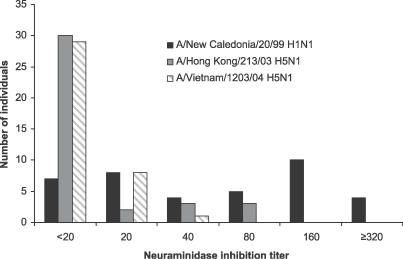
Reactivity of Human Donor Sera with NA of H1N1 and H5N1 Influenza Viruses Serum samples from human donors were analyzed by NA inhibition assay for reactivity with the NA proteins of A/New Caledonia/20/99 (H1N1), A/Hong Kong/213/03 (H5N1), and A/Vietnam/1203/04 (H5N1).

We also tested serum samples from human volunteers for reactivity with avN1. Analysis of these samples by NA inhibition assay demonstrated reactivity with H1N1 influenza virus A/New Caledonia/20/99 in 31 of 38 individuals tested (Figure 5). NA inhibition titers in these samples ranged from less than 20 to 320 or higher. We detected low inhibitory activity (titers between 20 and 80) against the NA of A/Hong Kong/213/03 in eight individuals and against the NA of A/Vietnam/1203/03 in nine individuals. Of these H5N1–reactive samples seven had reactivity to both H5N1 viruses. Although this human donor sample size is small, the results establish that some individuals have functionally significant levels of avN1-reactive antibodies.

## Discussion

The common NA subtype between avian H5N1 and human H1N1 influenza viruses raises the possibility that H1N1-specific immunity could offer a degree of protection against lethal H5N1 infection. Precedent for this possibility is found in the onset of the 1968 Hong Kong influenza pandemic. With respect to HA, the H3N2 virus responsible for this outbreak was antigenically novel, but in terms of NA it could not be distinguished from preceding H2N2 strains that descended from the 1957 Asian influenza pandemic virus [16]. The H3N2 virus of 1968 was generally less lethal than previous pandemic viruses, though the severity of disease varied across regions of the globe [17,18]. It has been proposed that NA-specific immunity against H2N2 virus moderated the virulence of H3N2 virus in humans. Evidence for this has been provided by epidemiological investigation [19], a human H2N2 vaccine study [20], and mouse prime/challenge experiments [16]. Similarly, it was previously shown that immunity raised against NA of a human H3N2 isolate by DNA vaccination partially protects mice against lethal challenge with an antigenically variant H3N2 virus [7].

Because H5N1 influenza virus has caused severe illness in most known human infections despite the common occurrence of H1N1 infection in the human population, it could be argued that immunity to huN1 is irrelevant in the face of H5N1 infection. However, known human cases worldwide are only a small sample set; a large number of mild cases may have gone undetected. Unreported mild or asymptomatic infection may be a frequent outcome of H5N1 exposure in persons with strong H1N1 immunity. Serologic studies to address this possibility would be valuable. The hypothesis that huN1-induced immunity confers some degree of protection against H5N1 virus implies that younger people, having a shorter history of H1N1 exposure, may be disproportionately susceptible to H5N1 infection. Consistent with this hypothesis are findings from an analysis of 144 cases of human H5N1 infection since 2003, reported by the World Health Organization: 50% of infected patients were younger than 18 years and 90% were 37 years or younger (Influenza Report, http://www.influenzareport.com).

Our data demonstrate in a mouse model that the immune response induced by the NA of human H1N1 influenza virus constitutes a modest defense against challenge with lethal doses of either PR8-avN1 or the H5N1 virus A/Vietnam/1203/04. There was a disparity between the proportion of mice that possessed detectable levels of cross-reactive IgG prior to infection (12.5%) and the rate of cross-protection against mortality (50% for A/Vietnam/1203/04). Because we later observed that mice passively immunized with serum from vaccinated animals were protected to a similar degree, it appears that antibodies play a dominant role in cross-protection. The IgG detection ELISA utilized here may lack the sensitivity to differentiate antibody from other inhibitory serum factors at low dilutions. Alternatively, cross-protective antibodies could be IgM or IgA.

These results suggest that immunization against H1N1 influenza virus with trivalent vaccines containing NA protein or via natural H1N1 infection can provide humans with some degree of resistance to H5N1 influenza viruses. Because most human influenza vaccines are inactivated, they are likely to elicit a predominantly humoral immune response. In our murine model, humoral immunity to huN1 is sufficient to mediate partial cross-lineage protection against H5N1 influenza. This finding underscores a possible benefit of seasonal influenza vaccination for a human population faced with the threat of pandemic H5N1 influenza and, more immediately, urges that emphasis be placed on increasing human seasonal influenza vaccine usage in areas where H5N1 is endemic in avian species. However, taking advantage of the potentially cross-protective property of NA may require that the antigenic content of both surface glycoproteins, not only HA, be standardized in licensed vaccines.

## Abbreviations

avN1: neuraminidase of avian-derived H5N1 influenza virus A/Vietnam1203/04
HA: hemagglutinin
huN1: neuraminidase of human H1N1 influenza virus A/New Caledonia/20/99
MLD_50_: 50% mouse lethal dose
NA: neuraminidase
PR8: influenza virus A/Puerto Rico/8/34 (H1N1)
PR8-avN1: reassortant PR8 virus in which the native NA has been replaced with avN1
PR8-huN1: reassortant PR8 virus in which the native NA has been replaced with huN1

## References

[pmed-0040059-b001] Wong SS, Yuen KY (2006). Avian influenza virus infections in humans. Chest.

[pmed-0040059-b002] Kida H, Webster RG, Yanagawa R (1983). Inhibition of virus-induced hemolysis with monoclonal antibodies to different antigenic areas on the hemagglutinin molecule of A/seal/Massachusetts/1/80 (H7N7) influenza virus. Arch Virol.

[pmed-0040059-b003] Yoden S, Kida H, Kuwabara M, Yanagawa R, Webster RG (1986). Spin-labeling of influenza virus hemagglutinin permits analysis of the conformational change at low pH and its inhibition by antibody. Virus Res.

[pmed-0040059-b004] Qiu M, Fang F, Chen Y, Wang H, Chen Q (2006). Protection against avian influenza H9N2 virus challenge by immunization with hemagglutinin- or neuraminidase-expressing DNA in BALB/c mice. Biochem Biophys Res Commun.

[pmed-0040059-b005] Johansson BE, Bucher DJ, Kilbourne ED (1989). Purified influenza virus hemagglutinin and neuraminidase are equivalent in stimulation of antibody response but induce contrasting types of immunity to infection. J Virol.

[pmed-0040059-b006] Johansson BE, Grajower B, Kilbourne ED (1993). Infection-permissive immunization with influenza virus neuraminidase prevents weight loss in infected mice. Vaccine.

[pmed-0040059-b007] Chen Z, Kadowaki S, Hagiwara Y, Yoshikawa T, Matsuo K (2000). Cross-protection against a lethal influenza virus infection by DNA vaccine to neuraminidase. Vaccine.

[pmed-0040059-b008] Johansson BE, Moran TM, Kilbourne ED (1987). Antigen-presenting B cells and helper T cells cooperatively mediate intravirionic antigenic competition between influenza A virus surface glycoproteins. Proc Natl Acad Sci U S A.

[pmed-0040059-b009] Powers DC, Kilbourne ED, Johansson BE (1996). Neuraminidase-specific antibody responses to inactivated influenza virus vaccine in young and elderly adults. Clin Diagn Lab Immunol.

[pmed-0040059-b010] Hoffmann E, Neumann G, Kawaoka Y, Hobom G, Webster RG (2000). A DNA transfection system for generation of influenza A virus from eight plasmids. Proc Natl Acad Sci U S A.

[pmed-0040059-b011] Salomon R, Franks J, Govorkova EA, Ilyushina NA, Yen HL (2006). The polymerase complex genes contribute to the high virulence of the human H5N1 influenza virus isolate A/Vietnam/1203/04. J Exp Med.

[pmed-0040059-b012] Selinsky C, Luke C, Wloch M, Geall A, Hermanson G (2005). A DNA-based vaccine for the prevention of human cytomegalovirus-associated diseases. Human Vaccines.

[pmed-0040059-b013] Webster RG, Campbell CH (1972). An inhibition test for identifying the neuraminidase antigen on influenza viruses. Avian Dis.

[pmed-0040059-b014] Yen HL, Monto AS, Webster RG, Govorkova EA (2005). Virulence may determine the necessary duration and dosage of oseltamivir treatment for highly pathogenic A/Vietnam/1203/04 influenza virus in mice. J Infect Dis.

[pmed-0040059-b015] Gao W, Soloff AC, Lu X, Montecalvo A, Nguyen DC (2006). Protection of mice and poultry from lethal H5N1 avian influenza virus through adenovirus-based immunization. J Virol.

[pmed-0040059-b016] Schulman JL, Kilbourne ED (1969). Independent variation in nature of hemagglutinin and neuraminidase antigens of influenza virus: Distinctiveness of hemagglutinin antigen of Hong Kong-68 virus. Proc Natl Acad Sci U S A.

[pmed-0040059-b017] Viboud C, Grais RF, Lafont BA, Miller MA, Simonsen L (2005). Multinational impact of the 1968 Hong Kong influenza pandemic: Evidence for a smoldering pandemic. J Infect Dis.

[pmed-0040059-b018] Kilbourne ED (2006). Influenza pandemics of the 20th century. Emerg Infect Dis.

[pmed-0040059-b019] Monto AS, Kendal AP (1973). Effect of neuraminidase antibody on Hong Kong influenza. Lancet.

[pmed-0040059-b020] Eickhoff TC, Meiklejohn G (1969). Protection against Hong Kong influenza by adjuvant vaccine containing A2-Ann Arbor-67. Bull World Health Organ.

